# Axonal Health Protects Against Cognitive Decline and Dementia Risk in Prodromal Alzheimer’s Diseases

**DOI:** 10.1002/alz70861_108012

**Published:** 2025-12-23

**Authors:** Zhaoyuan Gong, Mustapha Bouhrara

**Affiliations:** ^1^ National Institute on Aging, Baltimore, MD USA; ^2^ Laboratory of Clinical Investigation, National Institute on Aging, Intramural Research Program, Baltimore, MD USA

## Abstract

**Background:**

As dementia rates in the U.S. are projected to rise sharply, there is an urgent need for objective, noninvasive biomarkers to detect and monitor early changes prior to the onset of Alzheimer’s disease (AD). Despite advances in biomarker research, more sensitive and specific tools are needed to characterize the heterogeneity, complexity, and progression of AD beyond the AT(N) framework. Emerging evidence suggests white matter degeneration, particularly axonal loss, may be a core feature of AD, yet imaging biomarkers that specifically quantify axonal density remain lacking.

**Method:**

To address this gap, we developed a novel MRI‐based biomarker, the axonal density index (ADI), derived from standard diffusion MRI (dMRI). ADI generates whole‐brain white matter maps of axonal density. We analyzed longitudinal ADI values, cerebrospinal fluid (CSF) biomarkers of AD pathology, and cognitive data from the Alzheimer’s Disease Neuroimaging Initiative (ADNI) cohort. Participants were classified as cognitively normal (CN) or cognitively impaired (CI) at baseline. Linear mixed‐effects models were used to assess group differences, longitudinal changes, and associations between ADI and cognitive outcomes. Comparative analyses using CSF biomarkers were also conducted.

**Result:**

The CI group exhibited lower baseline ADI and a faster rate of decline over time compared to the CN group. In the CI group, higher baseline ADI was associated with slower cognitive decline and a reduced risk of dementia. Longitudinal reductions in ADI correlated with worsening cognition and increased dementia risk. Notably, ADI outperformed CSF biomarkers in predicting future cognitive outcomes.

**Conclusion:**

These findings highlight axonal degeneration as a key early feature of AD and suggest that greater axonal integrity may confer cognitive resilience. The axonal density index (ADI) offers a promising, noninvasive imaging biomarker that enhances early detection, refines the AT(N) framework, and enables more accurate monitoring of disease progression. This work advances the development of cost‐effective and personalized approaches to AD diagnosis and intervention.

